# 肺内多发小结节术前CT引导下微弹簧圈定位的初步探讨

**DOI:** 10.3779/j.issn.1009-3419.2018.11.08

**Published:** 2018-11-20

**Authors:** 凤卫 李, 应泰 陈, 建伟 边, 兴 辛, 思杰 刘

**Affiliations:** 100076 北京，北京航天总医院胸外科 Department of Thoracic Surgery, Beijing Aerospace General Hospital, Beijing 100076, China

**Keywords:** 计算机断层扫描, 定位, 肺肿瘤, 多发性, 肺切除术, Computed tomography (CT), Localization, Lung neoplasms, Multiple primary, Pneumonectomy

## Abstract

**背景与目的:**

肺内多发小结节微创手术的成功与否有赖于术前定位，然而目前缺乏针对肺内多发小结节术前定位的临床研究。本研究旨在与同期肺内单发小结节定位相比，探讨行电视胸腔镜手术（video-assisted thoracoscopic surgery, VATS）术前电子计算机断层扫描（computed tomography, CT）引导下留置微弹簧圈定位肺内多发小结节的临床价值。

**方法:**

回顾性分析术前行肺内小结节微弹簧圈定位者235例的临床资料。根据结节是否为单发分为：单发结节组184例（single nodule group），多发结节组51例（multiple nodules group）。单发结节组常规方式CT引导下定位，多发结节组在CT引导下分级、分批次留置微弹簧圈定位，统计分析两组定位成功率、并发症、病理结果及定位操作相关数据等。

**结果:**

多发结节组定位成功率达90.2%，与同期单发结节组成功率相比无统计学差异（90.2% *vs* 94.6%, *P*=0.205），多发结节组气胸发生率与单发结节组亦无统计学差异（21.6% *vs* 14.1%, *P*=0.179），然而多发结节组的操作时间明显长于单发结节组的操作时间[(30.6±6.6) min *vs* (19.9±7.4) min, *P*=0.000]。两组均无大咯血、空气栓塞及血胸发生等严重并发症。两组均无因术中无法定位结节而中转开胸者；手术方式以亚肺叶切除为主；术后病理以非浸润性病变为主。

**结论:**

对于需行胸腔镜手术的肺部多发小结节，按照一定策略，术前CT引导下分级、分批次留置微弹簧圈的定位方法安全、有效，值得推广。

## Abstract (en)

**Background and objective:**

Localization of multiple small lung nodules is the technical difficulty of minimally invasive operation resection. However, there are few clinical studies on the preoperative localization of multiple small lung nodules. This study was designed to evaluate the clinical value of preoperative computed tomography (CT) guided microcoil localization for multiple small lung nodules compared with single small lung nodule before video-assisted thoracoscopic surgery (VATS).

**Methods:**

A retrospective analysis of the clinical data of 235 patients with preoperative pulmonary nodules microcoil localization was performed. According to whether the nodules were single, they were divided into single nodule group (184 cases) and multiple nodules group (51 cases) (multiple nodules group). The single nodule group was positioned under CT-guided conventional methods. The multiple nodules group were CT guided localized by microcoil in batches according to priority before VATS. The success rate, complications, pathological results and localization operations related data were statistically analyzed.

**Results:**

The success rate of localization in multiple nodule groups was 90.2%, there was no significant difference compared with the single nodule group (90.2% *vs* 94.6%, *P*=0.205). The occurrence rate of pneumothorax in multiple nodule group and single nodule group was no statistical difference (21.6% *vs* 14.1%, *P*=0.179), however, the operation time in the multiple nodule group was significantly longer than the single nodule group [(30.6±6.6) min *vs* (19.9±7.4) min, *P*=0.000]. There were no serious complications such as massive hemoptysis, air embolism or hemothorax. There was no conversion to thoracotomy due to failure of localizing the nodules during operation. Sub-lobectomy was the main method of operation. The majority of postoperative pathologies were non-invasive carcinomas.

**Conclusion:**

For multiple small lung pulmonary nodules requiring thoracoscopic surgery, according to certain strategies, preoperative CT-guided localized by microcoil in batches according to priority before VATS is safe and effective, and worthy of promotion.

目前在全世界范围肺癌仍然是肿瘤相关死亡的首要因素。随着大气污染和人群健康意识的提高，计算机断层扫描（computed tomography, CT）筛查越来越广泛地应用于临床，肺癌的发生率和检出率逐年提高^[1]^。肺癌的CT表现多为孤立性肺结节，学术界对于孤立性肺结节的认识已越来越充分^[2]^。近年来临床上肺内多发小结节的检出逐渐增多，既往通常将肺内多发小结节考虑为肺部转移瘤或者非肿瘤性病变而失去手术机会，随着认识水平的提高人们发现部分患者为多原发肺癌，手术治疗效果与早期肺癌相似，故而部分多发小结节需要手术治疗^[3, 4]^。手术原则为完整、完全、迅速切除病灶的同时尽可能保留健康肺组织，手术方式首选胸腔镜手术^[5]^。肺内小结节胸腔镜术中仅靠触诊难以定位，触诊失败率为63%-88%^[6, 7]^，单发结节定位失败时术者往往采取扩大切除范围甚至直接切除结节所在肺叶，这种盲目扩大切除范围的方式显然不适用于多发结节，加之多发结节患者一次手术切除不完全也必将术者带入困境，因此肺内多发小结节手术的成功与否更是有赖于术前定位^[6]^。目前缺乏针对肺内多发小结节术前定位的临床研究，我们采用术前CT引导下分批、分次留置微弹簧圈定位肺内多发小结节，取得较好的临床效果，现将结果报道如下。

## 1 资料与方法

### 1.1 临床资料

纳入标准：临床可疑为恶性病变者；无肺外远处转移者；病变小、密度低无法触及或无法全部触及者。肺内病变需满足以下影像学标准：①纯磨玻璃影或实性成分≤1 cm的非纯磨玻璃影；②直径≤1 cm的实性结节或实性成分≥1 cm的非纯磨玻璃影，结节的实性成分与脏层胸膜距离≥0.5 cm。排除标准：直径≤0.4 cm的纯磨玻璃影不予定位及切除；严重心、肺功能不全；凝血机制障碍等。

2015年2月-2017年10月间，北京航天总医院胸外科共进行肺结节手术846例，因病变较小术前行微弹簧圈定位者235例。根据结节是否为单发分为：单发结节组184例，多发结节组51例。其中多发结节患者51例共143枚结节，定位126枚结节，两组患者一般情况见表 1。

**1:** 51例患者的一般情况 Clinical characteristics of 51 patients

Variables	Multiple nodules group (*n*=51)	Single nodule group (*n*=184)	*P*
Age (yr)	58.9 (34-81)	62.5 (27-84)	0.092
Gender			0.101
Male	17 (33.3%)	85 (46.2%)	
Female	34 (66.7%)	99 (53.8%)	
Number of localized nodules	126	184	0.000
1	0	184	
2	35	0	
3	11	0	
4	3	0	
5	1	0	
6	1	0	
Diameter of nodules (Mean±SD, mm)	8.2±3.8	8.8±2.7	0.570
Density of nodules			0.774
Pure GGO	70 (55.5%)	107 (58.2%)	
Mixed GGO	33 (26.2%)	49 (26.6%)	
Solid nodules	23 (18.3%)	28 (15.2%)	
Distance from nodule to pleura (Mean±SD, mm)	10.7±8.7	10.9±6.5	0.944
GGO: ground-glass opacity.			

### 1.2 设备与材料

CT机（美国GE Lightspeed 16排螺旋CT），CT定位标尺，穿刺针（22 G，美国Angiotech），微弹簧圈（COOK Microcoil金属丝直径0.018''，长度7 cm-9 cm），10 mL注射器，2%利多卡因。

### 1.3 穿刺方法

#### 1.3.1 多发结节组

根据结节数量、大小、优先级、手术方式、结节和预计切口关系，规划拟穿刺结节的顺序、穿刺体位。首先选择第一穿刺优先级结节（可以为多个结节，位于相同扫描野，相同体位可暴露）所需的体位，粘贴CT定位标尺，进行扫描，选择最佳穿刺点，测量拟穿刺点皮肤至壁层胸膜间的深度即胸壁软组织厚度，测量穿刺点至微弹簧圈留置靶位置的深度、进针角度，常规消毒铺巾，2%利多卡因局麻胸壁软组织，以22 G穿刺针按照所测量角度和深度刺入胸壁软组织，注意不能刺破壁层胸膜。如第一穿刺优先级结节为多个，同法进行其余结节的胸壁穿刺。重复CT扫描，如穿刺针的位置和角度满意，则将穿刺针针尖刺破胸膜推至靶位置；如CT扫描发现进针点、进针角度存在偏差，则予以调整，直至穿刺针位置、角度合适后，再进针将穿刺针针尖刺破胸膜推至靶位置。重复CT扫描，确认针尖位于靶位置，分别先于病灶周围释放部分微弹簧圈，缓慢退针至壁层胸膜下再次释放剩余部分微弹簧圈（如同体位定位多个结节，为尽量减少射线暴露，同时定位-调整-释放所有微弹簧圈，而不是逐一重复操作）。完成操作后再次重复CT扫描确认效果。更换体位，同法行其余优先级结节穿刺定位。

#### 1.3.2 单发结节组

与多发结节组穿刺、定位法相同，特点为只穿刺、定位一个结节，无需更换体位。

### 1.4 手术方法

胸腔镜下全面探查胸腔，通过操作口进行触诊，结合定位影像确定结节的位置，必要时行术中X线协助，并以电凝钩烧灼标记病灶，按照结节的位置、大小、病变可能的病理性质、患者预期剩余寿命等综合确定切除方案，先以卵圆钳夹持微弹簧圈、结节以及周围少量正常肺组织，用内镜直切割缝合器楔形切除病灶送术中快速病理。根据快速病理结果、病变多少、病变部位、对侧肺内有无结节等决定下一步手术方案。

## 2 结果

### 2.1 两组患者定位相关情况

2015年2月-2017年10月间，多发结节组共51例患者，对126枚结节进行术前定位，平均定位时间30.6 min，46例患者成功（90.2%），5例微弹簧圈脱位；单发结节组184例患者，对184例患者进行定位，平均定位时间19.9 min，174例患者成功（94.6%），6例微弹簧圈脱位。通过比较发现，多发结节组定位成功率达90.2%，与同期单发结节组成功率相比无统计学差异（90.2% *vs* 94.6%, *P*=0.205），多发结节组气胸发生率与单发结节组亦无统计学差异（21.6% *vs* 14.1%, *P*=0.179），然而多发结节组的操作时间明显长于单发结节组的操作时间[(30.6±6.6) min *vs* (19.9±7.4) min, *P*=0.000]。两组脱位患者有血肿者可术中通过血肿确认病灶位置，微弹簧圈脱位后附着于壁层胸膜者，可经过充气鼓肺、肺内针孔和血肿成功确认病灶位置。两组患者均无严重并发症发生，轻微并发症主要为无症状气胸，无需行胸腔闭式引流者，患者定位情况见表 2。

**2:** 两组患者定位相关情况 Procedural related parameters

Variables	Multiple nodules group (*n*=51)	Single nodule group (*n*=184)	*P*
Time interval from localitation to surgery			0.077
On the day of surgery	5 (9.8%)	7 (3.8%)	
The day before of surgery	42 (82.3%)	168 (91.3%)	
2 days before surgery	3 (5.9%)	9 (4.9%)	
3 months before surgery	1 (2.0%)^*^	0	
Average procedural time (Mean±SD, min)	30.6±6.6	19.9±7.4	0.000
Success rate	46 (90.2%)	174 (94.6%)	0.205
Complication			
Asymptomatic pneumothorax	11 (21.6%)	26 (14.1%)	0.197
Patient with 1 nodules	-	26 (14.1%)	
Patient with 2 nodules	4/35 (11.4%)	-	
Patient with 3 nodules	3/11 (27.3%)	-	
Patient with 4 nodules	2/3 (66.7%)	-	
Patient with 5 nodules	1/1 (100.0%)	-	
Patient with 6 nodules	1/1 (100.0%)	-	
Severe chest pain	1 (2.0%)	2 (1.1%)	0.598
Hemoptysis	0	1 (0.5%)	0.623
Gas embolism	0	0	-
^*^The patient's electrocardiographic monitoring showed a depression in the ST segment of the electrocardiogram after induction of anesthesia, further myocardial myocardium enzyme examination confirmed acute myocardial infarction, the surgery was canceled, the surgery was postponed 3 months later when the cardiac condition was stable.			

### 2.2 两组患者手术及术后病理情况

两组患者均在胸腔镜下完成既定手术，无因结节定位困难而中转开胸者，手术方式以亚肺叶切除为主，多发结节组肺叶切除+楔形切除的比例多于单发结节组，而单纯肺叶切除的比例少于单发结节组，两组患者病理均以早期肺癌及癌前病变为主，具体见表 3。

**3:** 两组患者手术及术后病理情况 Surgical and pathological parameters

Variables	Multiple nodules group (*n*=51)	Single nodule group (*n*=184)	*P*
Surgical methods			0.000
Wedge resection	31 (60.8%)	128 (69.6%)	
Wedge resection+Lobectomy	15 (29.4%)	13 (7.1%)	
Lobectomy	3 (5.9%)	36 (19.6%)	
Nodule removal	2 (3.9%)	7 (3.8%)	
Postoperative pathology			0.416
Invasive carcinoma	15 (29.4%)	73 (39.7%)	
Minimally invasive adenocarcinoma (MIA)	7 (13.7%)	39 (21.2%)	
Adenocarcinoma in situ (AIS)	7 (13.7%)	19 (10.3%)	
Atypical adenomatoid hyperplasia (AAH)	7 (13.7%)	16 (8.7%)	
Inflammatory nodules	12 (23.5%)	25 (13.6%)	
Sclerosing pneumocytoma	0	3 (1.6%)	
Hamartoma	2 (3.9%)	6 (3.3%)	
Metastases	1 (2.0%)	3 (1.6%)	

## 3 讨论

近年来临床上多发肺结节的诊断比例逐渐增高，尤其是影像学表现为磨玻璃样病变的多发结节更为常见，我单位多发结节患者占全部肺结节患者的比例亦高达6%，与文献^[3]^报道相符。既往由于认识不足，对于多发结节一般采取观望态度。目前国际上针对多原发结节的诊治主要参考2013年美国胸科医师协会（American College of Chest Physicians, ACCP）第三版的肺癌诊治指引，近年来国内在此方面研究较快，国内外均形成了选择性积极干预的共识。然而多发肺结节在手术干预的过程中有其特殊性，比较突出的问题即为本研究所涉及的术前定位问题^[8-11]^。

国际上对肺内小结节穿刺定位的研究始于20世纪90年代^[6]^。而我国在此方面的研究起步较晚，最早的研究见于2011年的带钩钢丝定位法，本研究所涉及的微弹簧圈定位于2014年有一项32例的小样本报道，并且尚无专门针对多发结节穿刺定位的研究^[12, 13]^。目前对于肺内小结节的定位方法总体上可以分为两大类：术中定位法和术前定位法。①术中定位法包括视诊、触诊、术中超声、计算机导航技术。其中视诊、触诊对病灶大小、质地、术者经验要求高，成功率低，因此视诊、触诊一般作为其他定位方式的补充。而超声及计算机导航技术要求配备大型术中设备，成本高昂无法普及，故目前术中定位法应用较少。②术前定位法为手术前在CT的引导下于病变周围放置各种标志物，包括金属材料的带钩钢丝、螺旋金属丝、微弹簧圈；液体材料的美兰、碘油、稀硫酸钡、放射性核素等^[14-23]^。通过综合文献报道以及我们自身的探索，微弹簧圈因结构纤细、质地柔软、包裹有纤维丝，可及时有效地堵塞穿刺针眼故而有气胸发生率相对低的优点，因此我们在操作中将定位材料锁定为微弹簧圈，但是微弹簧圈的缺点在于价格昂贵，随着结节数量的增加这个缺点尤为明显。

我们通过术前CT引导下留置微弹簧圈定位肺部多发小结节，引导进一步胸腔镜下手术，成功率达90.2%，与同期单发结节组成功率相比无统计学差异（90.2% *vs* 94.6%, *P*=0.205），取得了满意的效果，也总结了肺部多发小结节的定位策略：①“摆位适床”：通常CT引导下肺结节定位患者多采用平卧位、俯卧位和侧卧位，非垂直方向的结节通过调整穿刺针的角度进行穿刺，角度不易调整，操作难度大。对于此类情况，我们的策略是将患者的位置进行调整，即患者与扫描床呈一定角度，使进针角度为垂直于水平面的方向，降低操作难度。②“排兵”有先后：因为多发结节的定位很难保证100%成功，操作前一定与术者商议优先次序，基本按照本次手术的目的，必须解决的结节优先定位，肯定无法触及的结节优先定位。③“布阵”分批次：为减少患者的射线暴露量，缩短操作时间，同时也可降低并发症，将可以同一体位下操作的结节同时定位-调整-释放所有微弹簧圈，而不是逐一重复操作。④“打擦边球”：对于结节临近重要脏器穿刺风险高，或直接穿刺路径有遮挡者，可将微弹簧圈放置于予以楔形切除的结节的前方和深面，这样如果切除了微弹簧圈，结节也就肯定切除了。⑤“间于齐楚”：对于结节相对集中（间距≤2 cm）的情况，无需逐个定位，可将微弹簧圈放置于结节中间的位置。⑥“示廓灯”定位法：对于较大的纯磨玻璃密度影，尤其是高龄患者，因此类患者需尽快结束手术且即便病理结果为恶性，优选切除范围亦为亚肺叶切除，此时迅速且完全的楔形切除非常重要，我们的策略为在楔形切除路径的前后方均放置一微弹簧圈，切除了两个微弹簧圈则微弹簧圈之内包含的磨玻璃密度结节肯定也完整切除了。

两组病例定位失败患者多数为微弹簧圈脱位，11个脱位病例中，有4例患者穿刺针路径经过叶裂，7例穿刺针刺入肺实质深度 < 1 cm，故为避免微弹簧圈脱位而导致定位失败，穿刺路径应尽量避免经过叶裂，穿刺针刺入肺实质内应 > 1 cm。操作中如穿刺针自行向胸壁外回弹退针，则为穿刺针未穿破脏层胸膜、呼吸过程中脏层胸膜将穿刺针向外推挤所致，此时释放会导致微弹簧圈放置于脏层胸膜外，需进一步进针直至有突破感，再释放微弹簧圈于肺实质内。

定位操作中一旦发生气胸可引起患者胸闷、憋气症状，影响患者呼吸和氧饱和度，增加脱位的可能性^[12]^，更为重要的是对后续的穿刺定位带来巨大的困难，甚至导致无法定位成功，尽量减少气胸尤其是大量气胸的发生对于多发小结节的定位尤为重要。两组病例最常见的并发症均为无症状气胸，其中多发结节组气胸率为21.6%，与单发结节组无统计学差异（21.6% *vs* 14.1%, *P*=0.179），并且与文献报道的单发结节定位气胸率相似^[24, 25]^。分析可能的原因为：①微弹簧圈结构纤细、质地柔软、包裹有纤维丝，可及时有效堵塞穿刺针眼；②穿刺针、微弹簧圈均为22 G，无进气体的缝隙；③操作过程中患者一直处于平静呼吸状态避免了深吸气导致的气体进入胸腔；④局麻时需注意进针深度略小于胸壁软组织厚度，避免刺破壁层胸膜导致气胸影响进一步操作，壁层胸膜的麻醉主要靠局麻药的渗透性浸润，待麻醉充分后更换22 G穿刺针按照所测量角度和深度刺入胸壁；⑤穿刺针的角度调整过程均位于壁层胸膜外，尽可能减少刺破胸膜的次数；⑥一旦刺入肺内除非偏差太大，一般不再做调整，保证每个穿刺针针道都有微弹簧圈堵塞。一旦发生气胸，且尚未完全定位成功，则需将穿刺针退至壁层胸膜外抽吸排气后再行穿刺定位，此时如遇到无法刺破脏层的情况，则穿刺肺组织时嘱患者深吸气屏住气以提高肺脏层胸膜的张力以利穿刺，尽量提高后续的操作成功率。

穿刺过程中通常要求患者长时间固定于某特殊体位，从而带来不适感甚至出现压疮、神经损伤等并发症，穿刺定位结节数量的增多势必会延长操作时间，本研究亦显示多发结节组的操作时间明显长于单发结节组的操作时间[(30.6±6.6) min *vs* (19.9±7.4) min, *P*=0.000]，故而对于多发结节穿刺定位过程中需在满足穿刺操作前提下，应注意尽量选择患者舒适的体位、加强保护，以避免出现体位相关并发症。

多发结节的气胸并发症随着定位次数的增加而明显增加，当定位结节 > 5次时气胸发生率达到了100%（表 2）。因此我们建议同侧数量 > 5枚的实性结节则选取较大、形态不同、位于不同肺叶的4枚予以定位切除。实际操作中我们体会到对于同一肺叶进行4次以上楔形切除后所剩肺组织往往出现膨胀不全的情况，因此我们认为对于同一肺叶如需分别行4个以上楔形切除的，不宜一一定位后楔形切除，而直接行肺叶切除或仅分别定位并楔形切除4个以内的主要病灶，余小病灶予留后续随访观察（右肺中叶除外，右肺中叶此界值为需分别行2个楔形切除）。为避免同时双侧气胸，双侧多发结节一般分期分侧处理。

综上，对于需行胸腔镜手术的肺部多发小结节，按照一定策略，术前CT引导下分批、分次留置微弹簧圈的定位方法安全、有效，值得推广。
